# A Novel Deep Neural Network for Robust Detection of Seizures Using EEG Signals

**DOI:** 10.1155/2020/9689821

**Published:** 2020-04-07

**Authors:** Wei Zhao, Wenbing Zhao, Wenfeng Wang, Xiaolu Jiang, Xiaodong Zhang, Yonghong Peng, Baocan Zhang, Guokai Zhang

**Affiliations:** ^1^Chengyi University College, Jimei University, Xiamen 361021, China; ^2^Department of Electrical Engineering and Computer Science, Cleveland State University, Cleveland, Ohio 44115, USA; ^3^School of Electronic and Electrical Engineering, Shanghai Institute of Technology, Shanghai 200235, China; ^4^Department of Ultrasound, The First Affiliated Hospital of Xiamen University, Xiamen 361005, China; ^5^Faculty of Computer Science, University of Sunderland, Sunderland, UK; ^6^School of Software Engineering, Tongji University, Shanghai 201804, China

## Abstract

The detection of recorded epileptic seizure activity in electroencephalogram (EEG) segments is crucial for the classification of seizures. Manual recognition is a time-consuming and laborious process that places a heavy burden on neurologists, and hence, the automatic identification of epilepsy has become an important issue. Traditional EEG recognition models largely depend on artificial experience and are of weak generalization ability. To break these limitations, we propose a novel one-dimensional deep neural network for robust detection of seizures, which composes of three convolutional blocks and three fully connected layers. Thereinto, each convolutional block consists of five types of layers: convolutional layer, batch normalization layer, nonlinear activation layer, dropout layer, and max-pooling layer. Model performance is evaluated on the University of Bonn dataset, which achieves the accuracy of 97.63%∼99.52% in the two-class classification problem, 96.73%∼98.06% in the three-class EEG classification problem, and 93.55% in classifying the complicated five-class problem.

## 1. Introduction

Electroencephalogram (EEG) is a noninvasive, effective technique used in clinical studies to decode the electrical activity of the brain. EEG is one of the critical technologies to identify an abnormality of the brain, such as detecting epileptic seizures. Seizures are transient neurological dysfunctions caused by abnormal brain neurons and excessive supersynchronized discharges. The visual inspection of EEG for seizure detection by expert neurologists is a time-consuming and laborious process, and the diagnosis may not be accurate because of the massive amounts of EEG data and the discrepant clinical judgment standards of different neurologists [1, 2]. Therefore, scientific research on EEG-based automatic detection of epilepsy has attracted much attention.

Numerous algorithms have been proposed in the literature for automatic detection of epileptic seizures. These methods can be roughly classified into two categories: conventional methods and deep learning- (DL-) based methods. Thereinto, most of the traditional methods use hand-engineered techniques for feature extraction from EEG signals and then conjunct with classifiers to recognize. The Bonn University EEG database is widely used, which is publicly available and labeled as A, B, C, D, and E. Details of the dataset are described in a later section. There is much-published work using the Bonn dataset for epilepsy detection. They concern three main classification problems: the two-class seizure detection problem focuses on the classification between nonseizures and seizures; the three-class epileptic classification problem focuses on the grouping of three different EEG categories (normal, interictal, and ictal); and the five-class recognition problem focuses on the classification of five distinct types (A, B, C, D, and E).

In 2009, Ocak [3] proposed a scheme for detecting epileptic seizures based on approximate entropy and discrete wavelet transform (DWT) of EEG signals. This framework obtained an accuracy of 96% for two-class EEG classification. Moreover, Tzallas et al. [4] demonstrated the suitability of the time-frequency analysis (TFA) to classify EEG segments for epileptic seizures. The authors employed the artificial neural network (ANN) as the classifier and achieved an accuracy of 100% for the two-class and three-class classification and 89% for the five-class case. In 2010, Subasi and Ismail Gursoy [5] employed principal component analysis, independent component analysis, and linear discriminant analysis to reduce the dimension of EEG signals, extracted statistical features from DWT, and then used support vector machine (SVM) for classification. This model yielded a seizure detection accuracy of 100% for two-class classification. In 2011, Orhan et al. [6] used the k-means algorithm to cluster from the wavelet coefficients and then classified a multilayer perceptron neural network (MLPNN). This model yielded maximum accuracy of two-class and three-class classifications that are 100% and 96.67%, respectively. In 2012, Acharya et al. [7] proposed a methodology for the automatic detection of normal, interictal, and ictal categories from EEG signals. They extracted four entropy features and then fed to a fuzzy classifier. This methodology achieved an accuracy of 98.1%. In 2014, Kaya et al. [8] used the one-dimensional local binary pattern (1-D-LBP) to extract features from raw EEG and, respectively, combined with five different classifiers, such as Bayes Net, SVM, ANN, logistic regression (LR), and functional tree (FT). The best-performing classifier was the Bayes Net classifier, which achieved 99.5% and 95.67% maximum accuracy for two-class and three-class classifications, respectively. The worst performing classifier was the LR classifier, which gained 96.50% and 66.67% maximum accuracy for two-class and three-class classifications, respectively. In 2015, Sharma and Pachori [9] proposed the features based on the phase space representation for the classification of epileptic seizure and seizure-free EEG signals. They employed the least squares support vector machine as a classifier, which gave 98.67% accuracy. In 2016, Sharmila and Geethanjali [10] studied the performance of the 14 different combinations of two-class epilepsy detection. They employed naive Bayes (NB) and k-nearest neighbor (KNN) classifiers for the derived statistical features from DWT, and the NB classifier obtained an accuracy of 100% in the classification of healthy eyes open and epileptic EEG data. In 2017, Zhang and Chen [1] employed local mean decomposition (LMD) to decompose raw EEG signals into several product functions (PFs) and then fed the features into five classifiers. The authors reported that the best-performing classifier was the SVM optimized by genetic algorithm (GA-SVM), and the average classification accuracy was equal to or higher than 98.1%. Bhattacharyya et al. [11] computed the Q-based entropy by decomposing the signal with the tunable-Q wavelet transform (TQWT) into the number of subbands and estimating K-nearest neighbor entropies (KNNE) from various subband cumulatively and used the support vector machine classifier with the wrapper-based feature selection method to be the classifier. This method achieved an accuracy of 100% and 98.6% of maximum efficiency for two-class and three-class classifications, respectively. Zahra et al. [12] presented a data-driven approach to classify five-class EEG classification using the multivariate empirical mode decomposition (MEMD) algorithm. And ANN was employed to be a classifier, which achieved 87.2% accuracy.

These conventional methods for the detection of seizures use hand-engineered techniques to extract features from EEG signals. And many of these traditional methods show good accuracy for one problem but fail in performing accurately for others [2]. For example, they identify nonseizure and seizure cases (the two-class classification problem) with excellent accuracy but show poor performance for the detection of three-class epilepsy classification. Deep learning is a new research direction of machine learning that automatically learns the inherent laws and features of sample data. As both the available data and computational ability of hardware continue to increase, deep learning has addressed increasingly complex applications with ever-increasing accuracy [13–15]. Recently, automatic detection of epileptic seizures based on deep learning methods received much attention.

In 2018, Acharya et al. [16] implemented a 13-layer deep convolutional neural network (CNN) algorithm to detect normal, preictal, and seizure classes. This model includes five convolutional (Conv) layers, five max-pooling layers, and three fully connected (FC) layers. On this three-class detection problem, it achieved accuracy, specificity, and sensitivity of 88.67%, 90.00%, and 95.00%, respectively. Moreover, Ullah et al. [2] proposed an automatic system for epilepsy detection based on an ensemble of pyramidal one-dimensional convolutional neural network models. The core component of the system is a pyramidal one-dimensional convolutional neural network (P-1D-CNN) model, which consists of three main types of layers: Conv, batch normalization (BN), and FC layers. The classification performance of the P-1D-CNN model is not very satisfactory. Hence, the authors introduced the majority-vote (M-V) module in the final stage of the P-1D-CNN model, which significantly improved the performance of the algorithm. In almost all the cases of two-class and three-class concerning epilepsy detection problems, it has given the accuracy of 99.1 ± 0.9%. In 2019, Turk and Ozerdem [17] obtained two-dimensional frequency-time scalograms by applying Continuous Wavelet Transform (CWT) to EEG records containing five different classes and used the CNN structure to learn the properties of the scalogram images. On all the two-class, three-class, and five-class classification problems involving seizures, its recognition accuracy is 98.5%∼99.5%, 97.0%∼99.0%, and 93.6%, respectively. Moreover, Hussein et al. [18] introduced a deep long short-term memory (LSTM) network to learn the high-level representations of different EEG patterns, using one FC layer to extract the most robust EEG features relevant to epileptic seizures. This model achieved 100% accuracy of the two-class, three-class, and five-class classification problems.

Despite the encouraging seizure detection results gained using the CNN models mentioned above, several improvements can still be achieved. First, some of these CNN models have relatively single model structures. The second issue is the small number of available samples, which is not enough to train a deep neural network model. As such, we felt motivated to develop a CNN model for detecting seizures efficiently with raw EEG signals. To address these issues, first, we add the BN layer and dropout layer into the traditional convolutional blocks for learning features, which may help in detecting seizures efficiently. Second, the segments of raw EEG were divided into many nonoverlapping chunks to increase the number of samples for training and test, which may help in using a small amount of available data for fully training a deep model. Research findings have shown that the proposed approach is advantageous in detecting seizures using EEG signals.

## 2. Materials and Methods

### 2.1. Description of EEG Dataset

**Figure 1:**
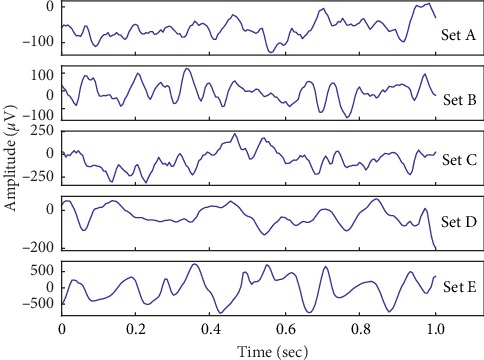
Sample EEG signals in this study.

Our seizure recognition experiments are conducted using the widely used and publicly available EEG database produced by Bonn University [19]. This database consists of five diverse subsets (set A–E) denoted as Z, O, N, F, and S. Sets A and B are composed of surface EEG recordings of healthy volunteers in the wakeful state with eyes open and eyes closed, respectively. On the other hand, Sets C, D, and E are gathered from patients with epilepsy. Thereinto, Sets C and D were recorded during seizure-free intervals. Set C was recorded from the hippocampal formation of the opposite hemisphere of the brain. Set D was recorded from within the epileptogenic zone. Set E only included seizure activities. Each of these sets contains 100 single-channel recordings of EEG signals with a sampling rate of 173.61 Hz and a duration of 23.6 s. The corresponding time-series is sampled into 4097 data points. Besides, the Rochester Institute of Technology divided every 4097 data points into 23 chunks. Each chunk contains 178 data points for 1 second (https://archive.ics.uci.edu/ml/datasets/Epileptic+Seizure+Recognition). To increase the number of samples for training a deep model, the Bonn dataset in this format is adopted, whose amount of sample increases 22 times. Therefore, the number of each category has 2300 EEG samples. Sample EEG signals of five EEG classes are shown in Figure 1.

### 2.2. Architecture of the Proposed Network

**Figure 2:**
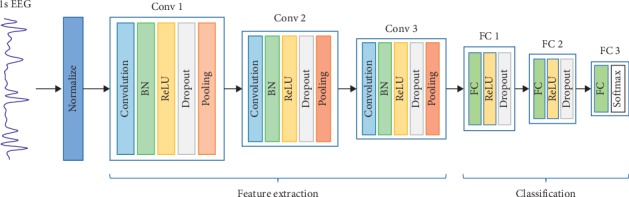
The proposed one-dimensional convolutional neural network architecture.

The deep CNN model [20] can automatically learn the features of EEG signals and performs classification in an end-to-end manner. The overall CNN architecture proposed in this paper is shown in Figure 2, which can perform feature extraction and classification. First, the input one-dimensional raw EEG data are normalized to zero mean and unit variance. Then, three convolutional blocks are adopted to learn features of the EEG signals, where each block consists of five layers. In detail, the first layer computes multiple convolutions in parallel to generate a set of linear activation responses. The second layer is BN, which is used to solve the internal variable shift. Each linear activation response passes a nonlinear activation function in the layer. The activation function used in this work is the rectified linear unit (ReLU) [21]. In the fourth layer, the dropout technology [22] is employed to prevent overfitting. The last layer of the block is the max-pooling layer, which introduces translation invariance. In the structure, the second and third convolutional blocks are similar to the first.

At the end of the third convolutional block, the feature maps are flattened into a one-dimensional vector that is connected to the FC layer for integrating features. The first two FC layers employ ReLU as the activation function, followed by a dropout layer. The third FC layer applies softmax as the activation function which will output a vector of probabilities corresponding to each category. To choose better model parameters, we explored eight models with different specifications. Details are described in the Experimental Results and Discussion section. In this study, we select model M7.  Table 1 shows the details of the proposed CNN structure.

### 2.3. Convolution Operation

A convolutional neural network (CNN) is a neural network designed to process data with similar network structures. The image can be regarded as a two-dimensional pixel grid. Similarly, time-series data can be considered as a one-dimensional grid formed by regularly sampling on time axis. The convolutional block of conventional CNN includes three layers: convolution, activation function, and pooling. For the one-dimensional EEG data used in this paper, the convolution operation is as follows:

(1) $$\begin{array}{c} s\left(t\right)=\left(x*w\right)\left(t\right)=\sum_{a}x\left(a\right)w\left(t-a\right). \end{array}$$

Convolution network has the characteristics of sparse interaction. So, it means fewer parameters need to be stored, which not only reduces the storage requirements of the model but also simplifies the calculation. At the same time, the parameters shared by the convolution kernel ensure that we only need to learn parameters that are many orders of magnitude smaller. Convolution is a kind of special linear operation, and activation function brings nonlinear characteristics into the network. The Rectified Linear Unit (ReLU) function is the most commonly used activation function in CNN, which overcomes the vanishing gradient problem, allowing models to learn faster and perform better. Equation (2) shows the ReLU function:

(2) $$\begin{array}{c} f\left(x\right)=\max\left\{0,x\right\}. \end{array}$$

The pooling function can reduce the spatial size of the representation to reduce the number of parameters and computation in the network. It replaces the output of the system at a specific position. For example, max-pooling gives the maximum value in several neighborhoods. The pooling can also help to make the representation approximately invariant to small translations of the input.

### 2.4. Calculation of BN

In this study, the BN layer and dropout layer are added to the traditional convolutional blocks. When training the deep neural network, the parameters of each layer are closely related to each other. The inconsistency in the distribution of layers' inputs causes a problem, called internal covariate shift. And the internal vary shift makes it difficult for us to choose an appropriate learning rate. To tackle this problem, Ioffe and Szegedy [23] developed BN technology which can almost reparametrize any deep networks, significantly reducing the problem of coordinated updates between multiple layers. The technology takes normalization as part of the model architecture and normalizes each mini-batch.

During training, BN calculates the sample mean and standard deviation for the mini-batch response H in backpropagation by

(3) $$\begin{array}{c} \mu =\frac{1}{m}\sum_{i}H_{i},\\ \sigma =\sqrt{\delta +\frac{1}{m}\sum_{i}{\left(H-\mu \right)}_{i}^{2}}, \end{array}$$

where the delta component *δ* is kept at a small positive value and is added only to avoid the gradient becoming undefined where the true standard deviation is zero. And they are used to normalize *H* by

(4) $$\begin{array}{c} H^{\prime }=\frac{H-\mu }{\sigma }. \end{array}$$

BN is also very useful in accelerating the convergence of the training phase and prevents overfitting. The technology has become a common practice, and the detail can be found in [23]. Therefore, we employ BN after every convolutional layer.

### 2.5. Feature Fusion and Classification

A deep neural network needs to learn a large number of parameters, which is likely to cause overfitting in the case of a small dataset. To address this issue, the authors [22] developed dropout technology to prevent the coadaptation of feature detectors. The critical idea of dropout is to randomly drop units with a predefined probability (along with their connections) from the neural network during training. It significantly reduces overfitting and gives significant improvements over other regularization methods. In the proposed model, we add the dropout lay after each ReLu activation function.

The output of the last convolutional block represents high-level features in the EEG signals. The fully connected layer is a usual manner of learning nonlinear combinations of these features. All the neurons in the last max-pooling layer are connected with all the neurons of the first FC layer. We used three FC layers. The number of neurons in the final FC layer (FC3) relies on the detection problem, e.g., for the two-class, three-class, and five-class epileptic classification problem, the number of neurons in FC3 is 2, 3, and 5, respectively.

The softmax activation function is a generalization of the binary form of logistic regression. It is commonly applied to the last layer of a deep neural network for constituting a categorical distribution over class labels and obtaining the probabilities of each input element belonging to a label. The softmax function, denoted by *h*_*θ*_(*x*^(*i*)^), is defined as equation (5), which represent the respective probabilities of the *i*-th sample (denoted by *x*^(*i*)^) belonging to each category:

(5) $$\begin{array}{c} h_{\theta }\left(x^{\left(i\right)}\right)=\left[\begin{array}{c} p\left(y^{\left(i\right)}=1\left|x^{\left(i\right)};\theta \right.\right)\\ p\left(y^{\left(i\right)}=2\left|x^{\left(i\right)};\theta \right.\right)\\ \vdots \\ p\left(y^{\left(i\right)}=k\left|x^{\left(i\right)};\theta \right.\right) \end{array}\right]=\frac{1}{\sum_{l=1}^{k}e^{\theta_{l}^{T}x^{\left(i\right)}}}\left[\begin{array}{c} e^{\theta_{1}^{T}x^{\left(i\right)}}\\ e^{\theta_{2}^{T}x^{\left(i\right)}}\\ \vdots \\ e^{\theta_{k}^{T}x^{\left(i\right)}} \end{array}\right], \end{array}$$

where *θ*_1_, *θ*_2_,…, *θ*_*k*_ are the softmax model parameters.

### 2.6. Training of CNN Model

Training the proposed model needs the weight parameters to be learned from the EEG data. For learning these parameters, we employed the conventional backpropagation algorithm with cross-entropy as the loss function. And, we used the stochastic gradient descent method with Adam optimizer that is based on the adaptive estimation of first-order and second-order moments. The hyperparameters of Adam algorithm are as follows: learning rate (0.0005), beta1(0.9), and beta2(0.999). The model was implemented in Keras, a powerful deep learning library, which runs on top of TensorFlow. The batch size of 100 is chosen in this work, which is used for each training update. To compare the performance measure, we trained all the models that are present in this work with 300 epochs.

### 2.7. Performance Measures

For evaluation, we adopted well-known performance metrics, such as accuracy (Acc), precision (Pre), sensitivity (Sen), and specificity (Spe), F1. Thereinto, accuracy is one of the most commonly used metrics in the literature, and it is defined as a ratio between the correctly classified samples to the total number of samples. The definitions of these performance metrics are as follows:

(6) $$\begin{array}{c} \text{Acc}=\frac{\text{TP}+\text{TN}}{\text{TP}+\text{TN}+\text{FP}+\text{FN}},\\ \text{Pre}=\frac{\text{TP}}{\text{TP}+\text{FP}},\\ \text{Sen}=\frac{\text{TP}}{\text{TP}+\text{FN}},\\ \text{Spe}=\frac{\text{TN}}{\text{FP}+\text{TN}},\\ \text{F}1=\frac{2\times \text{Pre}\times \text{Sen}}{\text{Pre}+\text{Sen}}, \end{array}$$

where TP (true positive) is the number of abnormal EEG records, which are correctly identified as abnormal; TN (true negative) is the number of normal EEG cases that are correctly predicted as normal; FP (false positive) is the number of normal EEG cases that are predicted as abnormal; and FN (false negative) is the number of abnormal EEG records that are incorrectly classified as normal.

To reduce the statistical uncertainty of test error estimation caused by small-scale test datasets, we adopted 10-fold cross-validation for evaluation. The 2300 EEG signals of each category are randomly divided into ten nonoverlapping fold. During the *i*-th test, the *i*-th fold of the EEG signals is used for testing while the remaining 9 folds are used for training. The accuracy, sensitivity, and specificity values reported in the paper are the average values obtained from ten evaluations.

## 3. Experimental Results and Discussion

Datasets are grouped with different combinations for exploring a general classification model, which is classified into two classes (nonseizures and seizures), three categories (normal, interictal, and ictal), and five classes (A, B, C, D, and E). To choose better model parameters, we considered eight models with different configurations.

### 3.1. Selection of Model

We explored models with different parameters, including the size of the receptive field, the number of neurons, and the dropout probability of the FC layer, for comparison. Taking the five-class classification problem, for example, the experimental results using 10-fold cross-validation are shown in Table 2.

Experiments show that within experimental parameters, a larger size of the receptive field and more neurons in the FC layer make the recognition more effective. The dropout probability of 20% in the FC layers is more effective than a rate of 50%. Therefore, the parameters of the model M7 with the best performance are used for experiments of two-class and three-class classifications with various combinations.

### 3.2. Performance of the Proposed Model

A multiple classification problem can be decomposed into multiple binary classification problems. The result of each classification can be listed as a confusion matrix, which reflects the original and predicted labels of each category.  Table 3 shows the confusion matrix and evaluation metrics of classification normal (B) vs. preictal (D) vs. seizure (E), as well as the overall classification result. All the metrics are over 96%, especially the specificity, which is above 98% in each category, and the overall classification.

To check the robustness of the proposed model, we tested 20 combinations. The detail of 10-fold cross-validation results is shown in Table 4, in which the average accuracy is employed as overall accuracy. The accuracy of the two-class classification varies from 97.63% to 99.52%, which has the best performance for A vs. E and the worst performance for D vs. E. The accuracy of the three-class recognition problem is between 96.73%∼98.06%. Notably, the accuracy is as high as 98.06% for B vs. D vs. E. The five-class classification problem is more complicated and harder to solve than the two-class and three-class problems but has an advantage in numerous clinical applications, and the proposed model still obtains an overall accuracy of 93.55%. The proposed model is suitable for various classification problems of the Bonn dataset and has a strong generalization ability.

### 3.3. Comparisons with Previous Studies

Numerous approaches have been presented in the literature for automated detection of epileptic seizures using the Bonn EEG database.  Table 5 shows the results of the comparison of the recognition rate of this work with them on various classification problems. The binary classification problem is the problem of identifying nonseizures and seizures. Classification of healthy volunteers and seizures is A vs. E, B vs. E, and AB vs. E. Due to the significant differences in this classification, the classification results of the various methods that appear in Table 5 are generally outstanding, all above 99%. The classification accuracy of interictal and ictal (C vs. E, D vs. E, and CD vs. E) is slightly lower than the first binary classification. In particular, both sets D and E are from the epileptogenic zone; therefore, it is difficult to distinguish. In the conventional methods of Table 5, Zhang et al. [1] obtained the best performance, which achieved 98.1% accuracy. In CNN-based technology, Ullah et al. [2] employed CNN and the majority-vote module to classify and gain 99.4% accuracy. Turk and Ozerdem [17] used CWT and CNN to recognize and achieved 98.50% accuracy. The proposed model of this work just employed CNN and obtained 97.63% accuracy.

The three-class classification problem further subdivides the EEG records to distinguish normal, interictal, and ictal EEG. We compared two types of three-class problem (B vs. D vs. E and AB vs. CD vs. E). The proposed model also achieved good performance. Especially in the case of B vs. D vs. E, its performance reaches the best accuracy of 98.06%, which is obviously better than another model [16] based on CNN only.

The five-class classification problem is more complicated and harder to classify than the two-class and three-class problems. It needs to identify the differentiation between EEG epochs belonging to the same class (e.g., sets A and B, which are both normal; sets C and D, which are both interictal). Therefore, in the literature, relatively some methods were proposed to address these three types of problems at the same time. The proposed CNN model achieved an accuracy of 93.55%, which is very close to the results of Turk and Ozerdem [17] and better than the conventional methods.

The experiment still needs to be implemented in reducing the learning rate and increasing the number of epochs, which will undoubtedly increase the accuracy of epilepsy recognition but, at the same time, will cost more time for training. For a limited number of training samples, we can also try to enhance the dataset, which may be useful for the generalization ability of the model. For example, we can divide the 23.6 seconds of EEG data into many overlapping chunks to further increase the number of samples.

## 4. Conclusion

A novel model for robust detection of seizures has been proposed, which deals with two-class, three-class, and five-class classification problems. The proposed approach has been developed based on the one-dimensional convolutional neural network model, which takes the raw EEG signal as input. To improve the learning ability of the model, the BN and dropout layers have been introduced to the traditional convolutional block. To address the issue of the small datasets, the EEG has been divided into many nonoverlapping chunks for training and test. The experimental result shows that the proposed model performs well on various EEG classification problems on the Bonn dataset.

## Figures and Tables

**Table 1:** Details of the CNN structure used in this research.

Block	Type	Number of neurons	Kernel size for each	Stride
		(Output layer)	Output feature map	
Conv 1	Convolution	139 × 20	40	1
	BN	139 × 20	—	—
	ReLU	139 × 20	—	—
	Dropout	139 × 20	—	—
	Max-pooling	70 × 20	2	2

Conv 2	Convolution	51 × 40	20	1
	BN	51 × 40	—	—
	ReLU	51 × 40	—	—
	Dropout	51 × 40	—	—
	Max-pooling	26 × 40	2	2

Conv 3	Convolution	17 × 80	10	1
	BN	17 × 80	—	—
	ReLU	17 × 80	—	—
	Dropout	17 × 80	—	—
	Max-pooling	9 × 80	2	2

FC 1	FC	64	—	—
	ReLU	64	—	—
	Dropout	64	—	—

FC 2	FC	32	—	—
	ReLU	32	—	—
	Dropout	32	—	—
FC 3	FC	2 or 3 or 5	—	—

**Table 2:** Configurations of 8 models using 10-fold cross-validation for the A vs. B vs. C vs. D vs. E cases.

Block	Parameter	M1	M2	M3	M4	M5	M6	M7	M8
Conv 1	Number of kernels	20	20	20	20	20	20	20	20
	Size of receptive field	5	5	5	5	40	40	40	40
	Dropout rate	0.2	0.2	0.2	0.2	0.2	0.2	0.2	0.2

Conv 2	Number of kernels	40	40	40	40	40	40	40	40
	Size of receptive field	3	3	3	3	20	20	20	20
	Dropout rate	0.2	0.2	0.2	0.2	0.2	0.2	0.2	0.2

Conv 3	Number of kernels	80	80	80	80	80	80	80	80
	Size of receptive field	3	3	3	3	10	10	10	10
	Dropout rate	0.2	0.2	0.2	0.2	0.2	0.2	0.2	0.2

FC 1	Number of neurons	32	32	64	64	32	32	64	64
	Dropout rate	0.2	0.5	0.2	0.5	0.2	0.5	0.2	0.5

FC 2	Number of neurons	16	16	32	32	16	16	32	32
	Dropout rate	0.2	0.5	0.2	0.5	0.2	0.5	0.2	0.5

A vs. B vs. C vs. D vs. E	Acc	90.47	88.61	91.89	91.20	93.37	91.62	**93.55**	92.92
	Sen	75.98	70.86	79.66	77.79	83.33	78.85	83.73	82.30
	Spe	94.00	92.72	94.92	94.45	95.83	94.71	95.93	95.57

**Table 3:** Confusion matrix for the three-class problem (B vs. D vs. E.) across 10-folds.

		Predicted			Acc	Sen	Spe	Pre	F1
		Normal	Preictal	Seizure					
Original	Normal	2263	36	1	98.32	98.39	98.28	96.63	97.50
	Preictal	49	2220	31	97.54	96.52	98.04	96.10	96.31
	Seizure	30	54	2216	98.32	96.35	99.30	98.58	97.45
Overall	—	—	*—*	—	98.06	97.09	98.54	97.10	97.09

**Table 4:** Accuracies (%) of 10-fold cross-validation using model M7.

Data sets combination	K1	K2	K3	K4	K5	K6	K7	K8	K9	K10	Mean
A vs. E	100	99.57	99.57	99.35	99.35	99.57	99.13	99.57	99.35	99.78	99.52
B vs. E	99.78	99.13	99.57	98.91	99.13	99.35	98.70	98.70	98.70	99.13	99.11
C vs. E	99.35	98.04	98.04	96.96	98.26	97.39	97.39	97.83	98.48	98.48	98.02
D vs. E	97.61	98.04	98.26	98.04	97.17	98.04	96.52	97.17	96.52	98.91	97.63
AB vs. E	99.57	99.13	99.57	99.57	99.57	99.13	99.57	99.13	99.57	98.99	99.38
AC vs. E	99.28	98.70	99.13	98.84	99.13	98.70	98.99	99.57	99.42	98.55	99.03
AD vs. E	98.12	97.83	98.41	98.70	98.41	98.41	98.55	98.55	99.13	98.84	98.50
BC vs. E	98.70	98.41	97.68	98.55	98.55	98.99	98.84	99.28	99.57	98.26	98.68
BD vs. E	97.39	97.10	97.54	98.84	98.26	97.54	98.41	97.97	97.83	97.39	97.83
CD vs. E	97.68	97.54	98.41	97.83	98.41	97.25	98.84	98.41	97.97	97.97	98.03
ABC vs. E	99.24	98.26	99.24	98.91	98.80	99.02	98.91	99.24	98.91	98.37	98.89
ABD vs. E	98.80	98.37	98.80	98.26	98.80	99.35	98.48	97.93	98.15	98.26	98.52
BCD vs. E	98.26	97.61	98.59	98.26	98.59	99.24	98.04	98.70	97.93	98.37	98.36
ABCD vs. E	98.96	99.22	98.70	98.52	98.35	99.22	98.78	98.61	99.13	98.09	98.76
A vs. C vs. E	96.04	97.05	97.00	97.39	94.98	97.58	97.00	96.09	96.81	97.39	96.73
A vs. D vs. E	97.63	97.10	97.54	95.94	97.00	96.67	97.39	97.87	96.81	96.43	97.04
B vs. C vs. E	97.63	97.97	98.12	97.68	98.36	97.20	97.87	99.03	97.68	97.58	97.91
B vs. D vs. E	98.35	98.30	98.07	97.49	98.26	97.97	97.20	98.45	98.45	98.06	98.06
AB vs. CD vs. E	96.70	97.10	97.74	96.43	96.72	97.97	94.96	97.91	96.96	97.25	96.97
A vs. B vs. C vs. D vs. E	92.99	94.37	94.00	93.41	93.36	92.73	93.74	93.25	93.74	93.91	93.55

**Table 5:** Comparison between the proposed method and other methods using the same dataset.

Data sets combination	Methodology	Study	Acc (%)	Our Acc (%)
A vs. E	TFA + ANN	Tzallas et al. [4]	100	99.52
	DWT + Kmeans + MLPNN	Orhan et al. [6]	100	
	1-D-LBP + FT/BN	Kaya et al. [8]	99.50	
	DWT + NB/KNN	Sharmila and Geethanjali [10]	100	
	TQWT + KNNE + SVM	Bhattacharyya et al. [11]	100	
	LMD + GA-SVM	Zhang and Chen [1]	100	
	CNN + M-V	Ullah et al. [2]	100	
	CWT + CNN	Turk and Ozerdem [17]	99.50	

B vs. E	DWT + NB/KNN	Sharmila and Geethanjali [10]	99.25	99.11
	TQWT + KNNE + SVM	Bhattacharyya et al. [11]	100	
	CNN + M-V	Ullah et al. [2]	99.6	
	CWT + CNN	Turk and Ozerdem[17]	99.50	

C vs. E	DWT + NB/KNN	Sharmila and Geethanjali [10]	99.62	98.02
	TQWT + KNNE + SVM	Bhattacharyya et al. [11]	99.50	
	CNN + M-V	Ullah et al. [2]	99.1	
	CWT + CNN	Turk and Ozerdem [17]	98.50	

D vs. E	1-D-LBP + FT/BN	Kaya et al. [8]	95.50	97.63
	DWT + NB/KNN	Sharmila and Geethanjali [10]	95.62	
	TQWT + KNNE + SVM	Bhattacharyya et al. [11]	98	
	LMD + GA-SVM	Zhang and Chen [1]	98.10	
	CNN + M-V	Ullah et al. [2]	99.4	
	CWT + CNN	Turk and Ozerdem [17]	98.50	

AB vs. E	DWT + NB/KNN	Sharmila and Geethanjali [10]	99.16	99.38
	CNN + M-V	Ullah et al. [2]	99.8	

CD vs. E	1-D-LBP + FT/BN	Kaya et al. [8]	97.00	98.03
	DWT + NB/KNN	Sharmila and Geethanjali [10]	98.75	
	CNN + M-V	Ullah et al. [2]	99.7	

ABCD vs. E	DWT + Kmeans + MLPNN	Orhan et al. [6]	99.60	98.76
	DWT + NB/KNN	Sharmila and Geethanjali [10]	97.1	
	TQWT + KNNE + SVM	Bhattacharyya et al. [11]	99	
	LMD + GA-SVM	Zhang and Chen [1]	98.87	
	CNN + M-V	Ullah et al. [2]	99.7	

B vs. D vs. E	CNN	Acharya et al. [16]	88.7	98.06
	CWT + CNN	Turk and Ozerdem [17]	98.00	

AB vs. CD vs. E	DWT + Kmeans + MLPNN	Orhan et al. [6]	95.60	96.97
	TQWT + KNNE + SVM	Bhattacharyya et al. [11]	98.60	
	LMD + GA-SVM	Zhang and Chen [1]	98.40	
	CNN + M-V	Ullah et al. [2]	99.1	

A vs. B vs. C vs. D vs. E	TFA + ANN	Tzallas et al. [4]	89	93.55
	MEMD + ANN	Zahra et al. [12]	87.2	
	CWT + CNN	Turk and Ozerdem [17]	93.60	

## Data Availability

The data used to support the findings of this study are available from the corresponding author upon request.
